# Using Heart Rate and Behaviors to Predict Effective Intervention Strategies for Children on the Autism Spectrum: Validation of a Technology-Based Intervention

**DOI:** 10.3390/s24248024

**Published:** 2024-12-16

**Authors:** Amarachi Emezie, Rima Kamel, Morgan Dunphy, Amanda Young, Heather J. Nuske

**Affiliations:** 1 Department of Psychology, University of Pennsylvania, Philadelphia, PA 19104, USA; aemezie@sas.upenn.edu; 2 Department of Psychiatry, Penn Center for Mental Health, Perelman School of Medicine, University of Pennsylvania, Philadelphia, PA 19104, USA; rima.kamel@pennmedicine.upenn.edu (R.K.); morgan.dunphy@pennmedicine.upenn.edu (M.D.); 3 Mayo Clinic, Mayo Eugenio Litta Children’s Hospital, Rochester, MN 55902, USA; young.amanda3@mayo.edu

**Keywords:** digital mental health, challenging behaviors, heart rate tracking, unobservable internal physiological data, observable behavioral data, intervention strategy effectiveness

## Abstract

Many children on the autism spectrum engage in challenging behaviors, like aggression, due to difficulties communicating and regulating their stress. Identifying effective intervention strategies is often subjective and time-consuming. Utilizing unobservable internal physiological data to predict strategy effectiveness may help simplify this process for teachers and parents. This study examined whether heart rate data can predict strategy effectiveness. Teachers and coders from the research team recorded behavioral and heart rate data over three months for each participating student on the autism spectrum using the KeepCalm app, a platform that provides in-the-moment strategy suggestions based on heart rate and past behavioral data, across 226 instances of strategy interventions. A binary logistic regression was performed to assess whether heart rate reduction, time to return to heart rate baseline, and documented skills and challenging behaviors predicted strategy effectiveness. Results suggested that heart rate reduction may be a significant predictor, and supported the existing practice of using behavioral patterns as proxies for strategy effectiveness. Additional analyses indicate proactive strategies are more effective and are associated with greater reduction in heart rate, relative to reactive strategies. Further exploration of how internal physiological data can complement observable behaviors in assessing intervention strategy effectiveness is warranted given the novelty of our findings.

## 1. Introduction

Autism spectrum disorder is defined as a disorder in which an individual exhibits persistent social communication and interaction differences across various contexts and displays limited and repetitive patterns of behaviors, interests, and activities [1]. Up to 80% of children on the autism spectrum exhibit one or more challenging behavior(s) [2]. Challenging behaviors are behaviors that are dangerous to the self or others and/or interfere with learning or development [3]. Some challenging behaviors that children on the autism spectrum display include aggression, self-injury, and property destruction [4]. Without appropriate intervention and support to reduce or prevent challenging behaviors, such behaviors may necessitate, in extreme cases, residential or inpatient care [5]. Given the prevalence of challenging behaviors in individuals on the autism spectrum and the potential impact on their environment, well-being, and care, it is important to identify and implement intervention strategies effective in managing challenging behaviors for each child, hence individualizing behavioral programming.

Proactive and reactive evidence-based intervention strategies are most commonly used in schools to manage challenging behaviors and promote emotion regulation [6,7,8]. Proactive strategies are initiated in anticipation of challenging behaviors in efforts to prevent such behaviors and/or promote skill acquisition [9]. These strategies include providing choices, tailoring the environment, and teaching functional communication [10]. In contrast, reactive strategies are implemented in response to challenging behaviors in efforts to minimize negative consequences and encourage positive behaviors [11]. These strategies may include extinction, redirection, and response interruption [12,13]. While both strategies are utilized in schools, proactive strategies are often favored over reactive strategies as it is best to avoid challenging behaviors from occurring all together. However, even when proactive strategies are properly implemented, challenging behaviors can persist [14,15,16].

Current practices in determining the effectiveness of an evidence-based intervention strategy for a given child rely heavily on traditional methods, i.e., tracking observable behaviors and skills, intervention strategies employed, and personal assessment of strategy effectiveness via pen and paper [17]. Educators are often tasked with observing, addressing, and recording such data, yet many do not receive the necessary training to refine behavioral plans or monitor how well a strategy works in addressing or preventing a challenging behavior [3].

Given the limitations of these time-consuming, labor-intensive, and subjective methods of assessing strategy effectiveness for each child, it would be better for educational teams to move toward a more objective, automated way of tracking strategy effectiveness. Previous research emphasizes the importance of identifying stress-related triggers in efforts to help educators effectively implement proactive intervention strategies centered on stress reduction and/or emotion regulation [3]. Therefore, it follows that evaluating changes in stress levels subsequent to the implementation of an intervention strategy may be crucial in assessing its effectiveness.

Measures of physiological stress may offer an objective, streamlined, and responsive approach to determining strategy effectiveness, with practical advantages over pen and paper analysis. One way to assess the physiological state of an individual is by measuring changes in their heart rate, an internal data measure [18]. Heart rate data can be easily recorded in real-time via wearable biosensors (i.e., heart rate trackers), which are widely commercially available and have been found to be comfortable for the majority of children on the autism spectrum [19]. Such data may then be used to provide reliable, real-time information to educators about when to implement proactive strategies.

In a series of studies, we and others have found that heart rate increase is associated with the onset of challenging behaviors in children on the autism spectrum. Our first study with preschoolers in a lab-based setting showed an average increase in heart rate of 22%, which was associated with oncoming behaviors (AUC = 0.72), on average, 58 s before the behavioral episode [20]. We replicated these results in a combined lab- and school-based study of elementary-school-aged children, finding, respectively, that a 27% and 29% average increase in heart rate (AUC = 0.75 and AUC = 0.75), on average, 72 and 80 s before, preceded the challenging behavior [21]. These results were also replicated in a dataset of children and adolescents (AUC = 0.71, 60 s before the behavioral onset) [22]. This relationship between heart rate increase, stress, and challenging behaviors implies that a reduction in heart rate after a challenging behavior may be associated with reduced stress and intervention strategy effectiveness. This translates to a potential opportunity to broaden the predictors used in evaluating intervention strategy effectiveness to incorporate physiological data.

One technology-based intervention that strives to do just that is KeepCalm—a smartphone application that uses observable behavioral data (such as challenging behaviors and skills acquired), alongside internal physiological data (heart rate), to determine the best intervention strategies for each student with autism [23]. KeepCalm aims to use heart rate data to better understand intervention strategy effectiveness, a departure from traditional approaches that rely heavily on subjective behavioral data alone [3].

This study used the KeepCalm app to investigate whether unobservable internal physiological data (heart rate) could be a predictor of intervention strategy effectiveness in addressing challenging behaviors of elementary-school-aged students on the autism spectrum. In this proof-of-concept study, we recruited teachers (*n* = 5) and trained them to collect and record student (*n* = 5) behavioral, heart rate, and interventional strategy data in a standardized fashion using the KeepCalm app. We hypothesized that, following the implementation of an intervention strategy, unobservable internal physiological data (heart rate) and observable behavioral data (subsequent challenging behaviors and skills) would serve as significant predictors of intervention strategy effectiveness. We also used this dataset to conduct exploratory analyses on the effectiveness of proactive vs. reactive strategies and intend to refine the KeepCalm “Top Strategies” algorithm based on our findings.

## 2. Materials and Methods

### 2.1. Materials

#### 2.1.1. KeepCalm App

KeepCalm is a digital mental health app that takes a collaborative approach to supporting emotion regulation in students on the autism spectrum. It uses interactive learning and data collection tools to help educational teams support students with challenging behaviors. The app syncs with wearable biosensors (i.e., heart rate trackers, accelerometers) to detect changes in physiological indicators of stress in students, notifying teachers in real-time when stress is heightened. Each notification includes recommended evidence-based strategies that can be used to mitigate heightened stress, encourage proactive coping, and prevent additional or continued challenging behaviors [3]. Key features within the platform include heart rate stress zone tracking, evidence-based resources, and pop-up notifications that provide in-the-moment clinical decision support.

**Figure A1:**
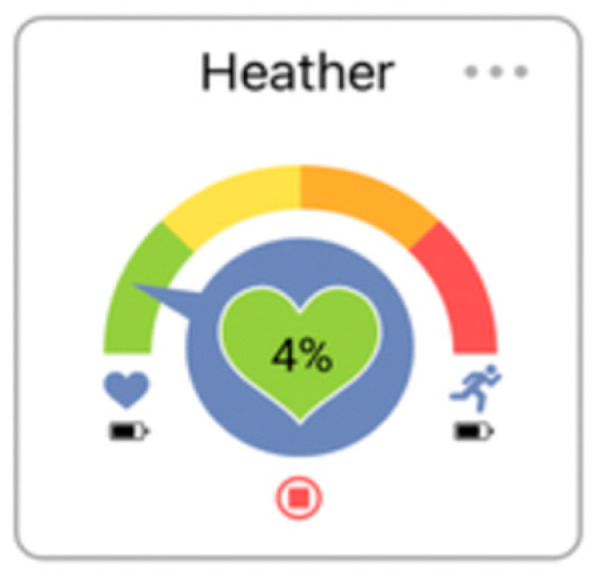
KeepCalm heart rate zone stress rainbow. Note. The arrow touching the heart rate stress rainbow indicates the color zone a given child is in with the percentage inside the heart indicating how a given child’s current heart rate compares to their baseline, e.g., in this image, the child Heather’s heart rate is 4% above their baseline.

The KeepCalm app categorizes heart rates via the stress zone rainbow depicted in Appendix A: Figure A1. The ‘green zone’ indicates typical heart rate at rest or baseline, the ‘yellow zone’ indicates a slight increase from this baseline (+10%), the ‘orange zone’ indicates a moderate increase (+20%), and the ‘red zone’ indicates a large increase (+30%). These zones were based on previous research into the association of heart rate increase and challenging behavior in children on the autism spectrum [20].

**Figure A2:**
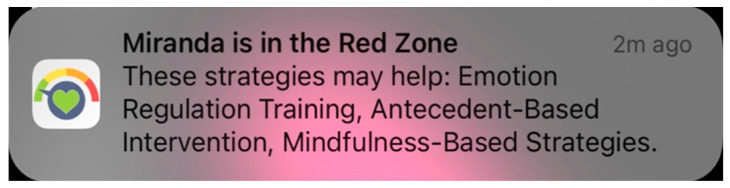
KeepCalm pop-up notification. Note. Example of pop-up notification educators receive when a child is in the orange or red heart rate zone.

When a student’s heart rate enters the ‘orange zone’ or ‘red zone’, it may signify an opportune moment for an educator to intervene and prevent challenging behaviors or support emotion regulation. KeepCalm notifies teachers and recommends three personalized strategies that, based on previous data collected by the app, support emotion regulation and mitigate the likelihood or continuance of challenging behaviors. These three “Top Strategies” are specified per child and per behavior. See Appendix A: Figure A2 for an example pop-up notification.

KeepCalm interventions are recommended from a collection of 27 proactive and reactive strategies that were derived from a systematic review of 95 studies on behavioral interventions for children with autism [10], which updated and extended the previous reviews in this area [4,14,24,25] and the 2020 National Clearinghouse on Autism Evidence and Practice (NCAEP) evidence-based practices guidelines [26]. Educators and parents have access to a full catalog of infographics and videos about these evidence-based intervention strategies through the KeepCalm app and website (https://www.digitalmentalhealth.org/keepcalm-resources (accessed on 1 September 2024)). This allows educational teams to enhance the ongoing professional development and in-class coaching that they receive from our center, even when their student is in the ‘green zone’.

The “Top Strategies” recommended to teachers during periods of heightened stress are determined by a rank ordering of data entered into the app, including heart rate, challenging behaviors, skills demonstrated, and the effectiveness of the intervention strategy used. An assessment of the data is carried out sequentially, progressing to the next criterion only if the preceding criteria is identical between strategies. See Table A2 in Appendix A for a detailed look at the criterion used to order rank strategies. One of the purposes of this study was to validate and refine this ranking algorithm.

#### 2.1.2. Heart Rate Trackers

We used two heart rate models in the study, the Mio Fuse wristband and Polar H7 chest-strap, both of which had undergone prior evaluation for accuracy, reliability, and comfort in children on the autism spectrum [19]. Both heart rate tracker models are highly accurate and reliable in measuring heart rate (HR) and heart rate variability (HRV). In evaluating the accuracy of the models through correlation with wired ECG, Mio Fuse demonstrated mean correlation of r = 0.91 with a [95% CI = 0.882–0.920], while the Polar H7 demonstrated a mean correlation of r = 0.99 with a 95% CI = 0.987–0.991 [19]. In assessing the reliability of the trackers, the Mio Fuse met the quality thresholds on spike rate 100% of the time, while the Polar H7 met the threshold 87.4–89.4% of the time. The sampling fidelity of the Mio Fuse was 96.2–97.1%, and the Polar H7’s sampling fidelity was 96.6–100% for HR/HRV measuring [19]. Teachers were provided with a Student Gadget Choice Guide visual support to help students decide which heart rate tracker they would like to wear.

#### 2.1.3. Accelerometer

To control for the heart rate increase associated with physical activity, a belt-clip accelerometer, mBient MetaMotionRL, measured the student’s movement. When a student engaged in high levels of movement—greater than or equal to 1 m per s squared (1 m/s^2^) for 5 s—they were placed in offline mode by the app. Offline mode temporarily pauses notifications about heart rate zone and strategy interventions for the teacher.

### 2.2. Methods

#### 2.2.1. Setting

This study was conducted in the School District of Philadelphia, the eighth largest public school district in the United States. For the last 16 years, our research team has partnered with the district, engaging in previous studies across more than 160 schools. Philadelphia teachers of autism support classrooms are provided with professional development and in-class coaching as part of our ongoing contract in efforts to improve access to evidence-based intervention for students on the autism spectrum. Autism support classrooms, classrooms that include only children with an educational classification of autism, provide specialized support for students on the autism spectrum. Such support includes structured routines, small-group instruction, visual supports, and class-wide management. The majority of the students in our study belonged to an autism support classroom.

#### 2.2.2. Participants and Recruitment

In this proof-of-concept study, the number of intervention strategies documented (rather than children) was most important (future studies are planned to examine this phenomenon across a larger sample of children). Five educational teams were recruited for this study in the context of a feasibility trial of KeepCalm [3]. Each team consisted of a student on the autism spectrum (no exclusion based on IQ or speaking ability) (*n* = 5), the student’s teacher (*n* = 5), and the student’s parent (*n* = 5).

#### 2.2.3. Procedure

The study was approved by the University of Pennsylvania IRB (829690) and School District of Philadelphia Research Review Committee (2022-12-1071). Teachers and parents from the School District of Philadelphia were invited to take a survey to determine their eligibility for our study. Eligible respondents were directed to one of two informed consents, depending on whether they were teachers or parents. Informed consent was obtained from all subjects involved in the study. After virtually signing the informed consent, participants were asked to complete a demographic survey. Both parents and teachers entered demographic information about themselves, while parents also provided demographic information for the student participant. Parents and teachers needed to share a student to be enrolled into the study.

After completing these surveys, educators and parents were directed to install the KeepCalm app on their personal iPhone. If an educator or parent did not have an iPhone, the research team provided them with one for the duration of the study. Additionally, each teacher received a heart tracker and accelerometer for use with the student participant during the study.

Upon receiving the necessary materials for the study, educators underwent training via a virtual intake meeting. During the intake meeting, educators were shown how to use the heart rate tracker and accelerometer and were instructed on how to assist their student in putting on these devices. Teachers were assisted in customizing the app for their student(s) by creating custom profiles for each participant and entering their specific triggers, behaviors/skills, and strategies. A default list of proactive and reactive evidence-based strategies used in supporting emotion regulation and mitigating challenging behaviors was pre-programmed in the app.

Recruited teachers were offered professional development and coaching on intervention strategies based on self-report data. Prior to testing, they were asked to self-assess their knowledge of 27 evidence-based proactive and reactive strategies on a 4-point scale, from “I don’t know this strategy” to “I know this strategy very well”. For each strategy they did not know, they were asked to indicate whether they were interested in attending a Zoom info-session with a member of the research team to review.

Additional support via infographics and videos on each strategy were accessible in the KeepCalm app for teachers (and parents) as needed. To further support teachers, both in-class device training and text support were available throughout the testing period.

Once the teacher completed training, the research team and education team calibrated the KeepCalm stress zone notification by establishing a baseline heart rate for each student participant. First, a research team member assisted the teacher in helping the student choose a preferred heart rate tracker (wristband vs. chest strap). Next, a baseline heart rate was recorded on the chosen device, during a 5 min period in which the student engaged in a relaxing activity involving little to no movement. To ensure accuracy, teachers were advised to measure 2–3 baselines. The lowest-recorded resting heart rate was considered the student baseline and used to establish the ‘green zone’.

The three-month testing period commenced after the baseline heart rate was recorded. Educators were instructed to place the heart rate tracker and accelerometer on the child and sync these devices with the KeepCalm app. This process was necessary for all in-class observations. During the testing period, student behavioral data were entered into the KeepCalm app by teachers as well as coders from the research team.

#### 2.2.4. Teacher Data Collection

During the three-month trial period, teachers were asked to track their use of reactive and proactive intervention strategies, the effectiveness of these strategies, and the accompanying behavioral data in the KeepCalm app, while the heart rate tracker simultaneously monitored student heart rate data [3]. Teachers were specifically encouraged to track skill and behavioral data in accordance with student Individualized Education Programs (IEPs)—educational programs of measurable goals—so that the information gleaned from the KeepCalm app could be used to modify and refine these living documents.

Heart rate trackers and accelerometers were used to determine when students entered the ‘orange’ or ‘red zone’ and notified teachers when students experienced heightened stress levels. Students in the ‘orange’ or ‘red zones’ were considered at risk of oncoming challenging behavior(s), and so, evidence-based interventions were recommended to the teacher via pop-up notifications, in an effort to reduce the likelihood of such behavior(s) occurring or continuing.

**Figure A3:**
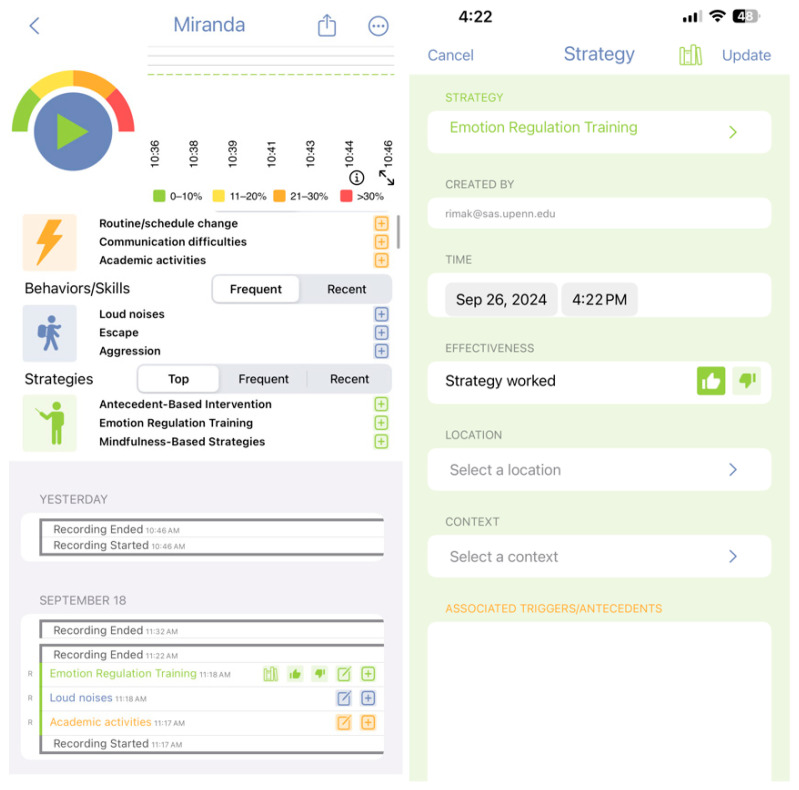
Teacher strategy data log form from KeepCalm.

Teachers were prompted by KeepCalm to log an observation each time they received an ‘orange’ or ‘red zone’ pop-up notification. They were asked to note challenging behavior(s), skill(s), intervention strategies used, and whether the strategy was effective. See Appendix A: Figure A3 for a screenshot the above-described teacher prompts.

If a teacher did not rate a strategy as effective or non-effective, or if no behaviors were recorded in the 3 min succeeding the implementation of a strategy, KeepCalm sent a pop-up notification reminding them to enter the success of the strategy. If no data were entered after this notification, the teacher was reminded to rate the strategy effectiveness whenever the app was opened or if another event was recorded.

Teachers were also encouraged to view daily reports, to export student data, and to communicate with parents through the app. To ensure fidelity, members of the research team reviewed teacher-recorded data, monitored usage of the app by educational teams, and performed in-class observations at least once per team over the course of the study.

#### 2.2.5. Research Team Data Collection

Coders on the research team were trained to observe and record student participant’s behavioral data. Coders first trained in reliability (a score of 80% or higher on three consecutive classroom video observations) before they could begin in-class coding. Upon meeting the reliability requirement, coders began live behavioral coding via in-class observations in which coders recorded behavioral IEP data. Coders were required to have a reliability score at or above 80% for two in-class observation before coding independently.

After reliability was established, coders on the research team started visiting each participant in school at least once for a 1 h in-class observation. Observations were scheduled when the teacher was likely to be giving instruction, when students are likely to engage in challenging behaviors. During each observation, heart rate was recorded through the KeepCalm app, while the coders recorded student behavioral data offline, so that it could be combined and compared with simultaneous teacher and heart rate data later.

Coders utilized the KeepCalm In-Class Observation Guide and Codebook to track behaviors, skills, interventions, and effectiveness. See Appendix A: Table A2 for a page from this codebook. They documented data on 4 × 15 min observation guides. Each 15 min observation guide was further subdivided into 3 min time intervals. Within these intervals, coders observed and recorded the behaviors of student participants (challenging behaviors and skill acquired), the intervention strategies used by educators (proactive and reactive), and the effectiveness of the intervention strategies (effective, not effective, and cannot be determined). Additionally, coders documented the time, the activity being engaged in, and any additional notes. See Appendix A: Table A2 for the 3 min interval data sheet from the observation guide. The behavioral data recorded on the observation guide aligned with behavioral data collection methods in KeepCalm app.

Throughout the data-recording process, coders had access to a codebook containing brief definitions of each challenging behavior, skill, proactive strategy, and reactive strategy. The codebook complemented the observation guide and worked to promote consistent coding practices.

## 3. Results

### 3.1. Data Analysis

Data collected by both the teachers and coders was used to determine if behavioral and heart rate data could be used to determine the effectiveness of an intervention strategy. We began our data analysis by reviewing descriptive statistics for the categorical and continuous variables of the study using the Kolmogorov–Smirnov test to analyze for skewness and outliers. Subsequent behaviors, subsequent skills, and minutes to return to green zone values were determined to be non-normal, but after further analysis we kept the original values to avoid biasing the results. Overall, we found that 24.05% of entries were missing strategy effectiveness data (i.e., a strategy was entered but not rated on effectiveness). Entries without strategy effectiveness were excluded from further analysis. Of the interventions with effectiveness ratings, 78.87% were rated effective while 21.13% were rated ineffective. Please see Appendix B: Table A3 for descriptive statistics about the effectiveness data.

During our analysis of descriptives, we identified outliers in heart rate reduction, as some entries reflected heart rate reduction by 100% (which would mean the heart was no longer beating). Upon further investigation, we learned that these outliers came from a child that removed their heart rate device during observation; therefore, these entries were also excluded from the dataset. After excluding these outliers, and removing observations with missing effectiveness data, 69.6% of entries (*n* = 179) were included in our final analysis.

We used a binary logistic regression to determine the significant predictors of effective vs. non-effective strategies, using four independent variables: two relating to heart rate data (time to return to heart rate baseline and magnitude of heart rate reduction) and two relating to behavioral data collection (children’s documented skills and challenging behaviors in the 5 min after a strategy was implemented). We then tested to see whether these results held when controlling for children.

Following this analysis, we conducted an exploratory investigation of the relationship between mean heart rate reduction, intervention strategy (e.g., reinforcement, role play), and strategy type (i.e., proactive vs. reactive). Trends in the descriptive statistics led us to perform an independent t-test, in order to assess whether mean heart rate reduction differed significantly between proactive and reactive intervention strategies. We then ran an additional binary logistic regression that included strategy type as a predictor variable for strategy effectiveness. All statistical analyses were executed using SPSS (Statistics V.29).

### 3.2. Predicting Strategy Effectiveness

To examine whether intervention strategy effectiveness could be predicted by both unobservable internal physiological data as well as observable behavioral data, we selected heart rate reduction, minutes to return to green heart rate zone, subsequent challenging behaviors, and subsequent skills as predictor variables. The descriptive statistics of these predictors are presented in Table 1. We performed a binary logistic regression to ascertain how these predictors influenced the likelihood of intervention strategy effectiveness, as presented in Table 2. The logistic regression model was statistically significant, χ^2^(4) = 27.11, *p* < 0.0005 and explained 23.3% (Nagelkerke R^2^) of the variance in strategy effectiveness. Heart rate reduction (*p* < 0.05), subsequent behaviors (*p* < 0.01), and subsequent skills (*p* < 0.01) were all found to be statistically significant in explaining strategy effectiveness, but minutes to return to green heart rate zone was not. According to these results, a 1% increase in heart rate reduction increases the likelihood of intervention strategy effectiveness by 6%. This outcome was not robust to the inclusion of children as a predictor; however, the model did not perform as well when controlling for children, and the step to include children was not statistically significant. Please see Appendix B: Table A4 for the Binary Logistic Regression.

### 3.3. Exploratory Analyses: The Association of HR Reduction with Intervention Strategy Type

**Figure 1:**
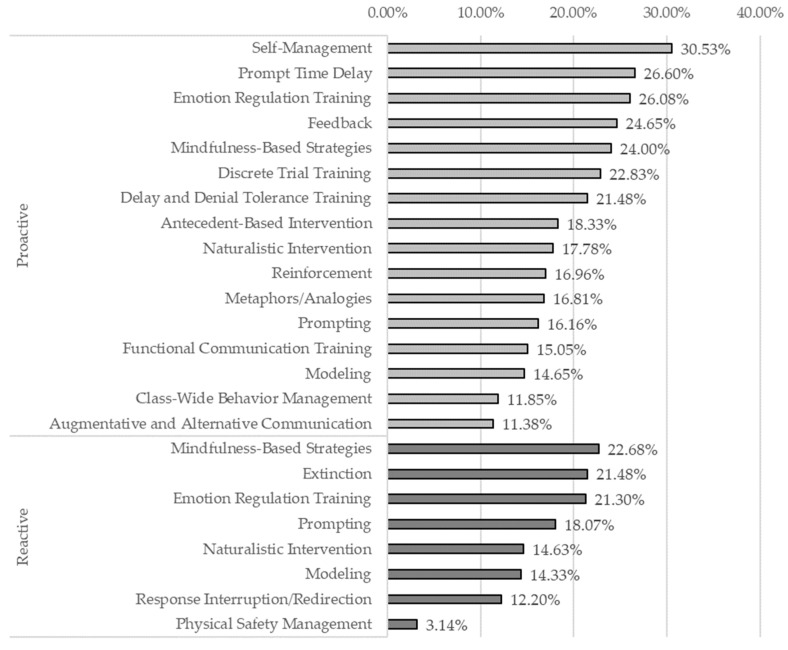
Mean heart rate reduction % across intervention strategies by strategy type.

After finding a significant relationship between heart rate and strategy effectiveness, we decided to explore the relationship between strategy type (i.e., proactive vs. reactive), heart rate reduction, and strategy effectiveness. Overall, 82.1% of observations recorded included a proactive strategy (*n* = 211), while 17.5% included a (*n* = 45) reactive strategy. One observation was coded for both and was excluded from this analysis. Of these observations, 87.9% had heart rate reduction data (*n* = 226) and were included in our dataset. Proactive strategies were rated effective 84.18% of the time, while reactive strategies were rated effective 55.56% of the time. An independent samples *t*-test was performed to evaluate whether there was a difference between the heart rate reduction in proactive strategies and reactive strategies. The results indicated that proactive strategies (*M* = 18.24, *SD* = 11.24) had significantly higher heart rate reduction than reactive strategies (*M* = 14.13, *SD* = 9.83), *t*(228) = 2.14, *p* = 0.033. We then looked at the magnitude of heart rate reduction by each intervention strategy under strategy type to see which strategies were driving these trends, displayed below in Figure 1. Note that the number of recorded uses of each strategy varied drastically, which may have influenced these figures. Please see Appendix B: Table A5 to see the frequency of intervention strategy by strategy type.

After finding a significant relationship between heart rate reduction and strategy type, we decided to test the explanatory power of strategy type on strategy effectiveness. We performed a binary logistic regression to ascertain how strategy type, heart rate reduction, minutes to green zone, subsequent behaviors, and subsequent skills influenced the likelihood of intervention strategy effectiveness, as presented in Table 3 below. The logistic regression model was statistically significant, χ^2^(5) = 33.82, *p* < 0.001 and explained 28.6% (Nagelkerke R^2^) of the variance in strategy effectiveness. Strategy type (*p* < 0.01), subsequent behaviors (*p* < 0.05), and subsequent skills (*p* < 0.01) were all found to be statistically significant in explaining strategy effectiveness; however, heart rate reduction (*p* = 0.101) and minutes to return to green heart rate zone were not. Results indicate that reactive strategies were 3.63 times more likely to be rated as effective when compared to proactive strategies ceteris paribus.

When controlling for children, strategy type and subsequent skills maintained their statistical significance, while subsequent behaviors did not. Like with our original analysis, the child was not found to be a statistically significant predictor for strategy effectiveness. Please see Appendix B: Table A6 for the results from this analysis.

## 4. Discussion

The main purpose of this study was to determine the predictive significance of unobservable internal physiological data and observable behavioral data on intervention strategy effectiveness, with the ultimate goal of validating and refining the “top strategy” algorithm within a technology-based intervention, KeepCalm. By incorporating physiological data into behavioral analysis for children on the autism spectrum, we broadened the scope of data traditionally used by educational teams to assess intervention strategy effectiveness. Our findings suggest that heart rate reduction may be a significant predictor of intervention strategy effectiveness. That is, when educators perceive their intervention strategies as effective, there is corresponding physiological regulation in children, as evidenced by a reduction in heart rate. The magnitude of the mean heart rate reduction observed further suggests that intervention strategies have a significant impact on reducing stress levels. Intervention strategies not only encourage positive behaviors in children but also contribute to their well-being.

These findings have the potential to enhance the reliability of assessing the effectiveness of intervention strategies through use of heart rate monitoring, which is more objective and requires less effort than behavioral data collection. We do not suggest that heart rate monitoring could or should replace behavioral and skills data collection altogether, but rather that it could be used to augment current practices. Traditional methods are cumbersome in nature and often difficult to carry out, given the demands of educators, since they rely exclusively on observable behaviors to determine intervention strategy effectiveness. Monitoring heart rate changes via biosensor is less burdensome to educators than pen and paper analysis and may save them time when incorporated into their data collection plan. By accounting for unobservable physiological changes, we hope to ultimately reduce the burden of data collection for educational teams and to expand their access to real-time, personalized, evidence-based recommendations.

Our findings bolster the efficacy of traditional methods by confirming that strategy effectiveness is significantly related to whether a child engages in skills and/or behaviors after an intervention. This indicates that behavioral data are an effective way to assess intervention strategy effectiveness. Of all predictors, subsequent skills had the most sizeable influence on intervention strategy effectiveness. That is, the predictive power of each additional skill acquired outweighed that of both challenging behaviors and heart rate reduction. This finding comes as a surprise as intervention strategies are primarily designed to prevent or manage challenging behaviors, with skill acquisition typically considered secondary [27]. This suggests that the effectiveness of intervention strategies may be more closely tied to skill acquisition than what research traditionally has anticipated. Future studies should look more closely at how skills acquired can be used to assess strategy effectiveness, since our findings suggest this predictor may be undervalued by current practices.

Moving beyond the parameters of our initial investigation, the exploratory analysis we ran on strategy types revealed that educators gravitated towards proactive strategy implementation over reactive strategy implementation, and that they tend to rate proactive strategies as more effective than reactive ones. Additionally, our results suggest that proactive strategies lead to a significantly greater reduction in heart rate compared to reactive strategies, indicating that students also felt physiologically better following the implementation of a proactive strategy. In conclusion, these findings suggest that there should be greater emphasis placed on implementing proactive strategies, a suggestion which aligns with current perspectives within the autism field [28].

More broadly, our findings corroborate existing research findings on the association of heart rate and stress levels. When an intervention strategy was implemented to reduce stress, the data showed a mean positive heart rate reduction. This relationship signals the importance of gaining a better understanding of the change in stress levels following the implementation of an intervention strategy, as well as the role of strategy type in determining effective intervention strategies.

All of these findings have implications for KeepCalm. Currently, the app monitors unobservable internal physiological data (heart rate) as well as observable behavioral data (challenging behavior and skills) to recommend “Top Strategies” suggested to educators based on a rank order. The rank order presently prioritizes predictors as follows: teacher ratings of strategy effectiveness, challenging behaviors subsequent to intervention, skills subsequent to intervention, heart rate reduction subsequent to intervention, minutes for a child to return to green heart rate zone, and time since the intervention was last used. Our results indicate that a reevaluation of these prioritized criteria is necessary. Subsequent skills may play a more significant role in predicting strategy effectiveness than its current rank implies. Based on the odd ratios Exp(B) identified in Table 3, skills acquired should be considered first in rank ordering, heart rate reduction second, challenging behaviors third, and minutes to return to green heart rate zone last.

Understanding the predictors of intervention strategy effectiveness is critical for addressing challenging behaviors in children on the autism spectrum. Looking beyond traditional predictors toward more automated, objective physiological methods of data collection is essential to improving the classroom experience for teachers and students alike. Our insights about the predictive role of heart rate in intervention strategy effectiveness is a step in that direction. Given the novelty of our findings, future research should delve deeper into how unobservable internal physiological data can best complement observable behavioral data when assessing intervention strategy effectiveness.

The KeepCalm app is amongst many apps starting to emerge in using heart rate to monitor symptoms or as a proxy for other clinical markers unfolding over time. For example, Shu et al. (2020) used heart rate data to index emotion in healthy adults [29], and Yang et al. (2024) use heart rate amongst other biosensors to examine a biometric signature in hospitalized depressed patients relative to healthy controls [30]. Arabian et al. (2023) studied the use of a digital platform integrating heart rate tracking to monitor patients’ emotional responses during therapy sessions so to give therapists real-time feedback so they may adjust their strategies or interventions in the moment [31].

While this study delivered valuable insights, it is important to acknowledge its limitations. First, even though the number of observations with heart rate data, skills, and behaviors was high (n = 226), the sample size of children observed was relatively small (n = 5). This limitation is currently being addressed in ongoing recruitment for the feasibility trial of KeepCalm in which a total of 30 students will be recruited.

Second, a minority of the data collected came from educators in the trial. This was due to the competing demands of teaching, as well as technical challenges with the KeepCalm app. Further research is necessary to determine how to streamline and simplify app data collection for teachers. To address this limitation, the KeepCalm development team is updating the app to incorporate educator feedback for an enhanced user experience and improved data collection process for future studies.

These limitations are important to acknowledge and address, but they do not take away from the novelty of our findings. There is no extant research that uses internal physiological data to predict intervention strategy effectiveness for children on the autism spectrum, and these early results look promising. Given the potential impact unobservable measures can have on quality of life for teachers and students alike, it is essential that further research validate and expand upon these findings.

## Figures and Tables

**Table 1:** Descriptive statistics of predictor variables.

Predictor	Mean	SD	Min	Max
HR ^1^ Reduction %	17.51	11.10	−21.67	61.85
Mins to Return to Green Zone ^2^	0.26	0.73	0	5.84
Subsequent Behaviors	0.88	1.69	0	8
Subsequent Skills	0.68	0.97	0	6

^1^ HR is an abbreviation for heart rate. ^2^ Green HR Zone is when a child’s heart rate is at baseline.

**Table 2:** Binary logistic regression on the effect of heart rate and behavioral data on intervention strategy effectiveness.

Predictor	Model	Summary	Individual Predictors			95% C.I. for Exp(B)	
	***p***	***χ^2^***	***p***	***Exp(B)***	***B***	***Lower***	***Upper***
*Block*	<0.001	27.11					
HR Reduction %			0.04	1.06	0.05	1.00	1.11
Mins to Return to Green Zone			0.45	0.82	−0.19	0.50	1.37
Subsequent Behaviors			0.004	0.73	−0.31	0.59	0.91
Subsequent Skills			0.001	3.75	1.32	1.72	8.18

**Table 3:** Binary logistic regression on the effect of strategy type, heart rate reduction % and behavioral data on intervention strategy effectiveness.

Predictor	Model	Summary	Individual Predictors			95% C.I. for Exp(B)	
	***p***	***χ^2^***	***p***	***Exp(B)***	***B***	***Lower***	***Upper***
*Block*	<0.001	33.82					
HR Reduction %			0.101	1.046	0.045	0.991	1.10
Mins to Return to Green Zone			0.354	0.783	−0.244	0.468	1.31
Subsequent Behaviors			0.036	0.784	−0.243	0.625	0.985
Subsequent Skills			0.001	3.65	1.29	1.67	7.94
Strategy Type (1)			0.009	3.63	1.29	1.38	9.58

*Note.* HR is an abbreviation for heart rate. Green HR Zone is when a child’s heart rate is at baseline. Strategy Type coded so that 0 is proactive and 1 is reactive.

## Data Availability

Data are available via direct request to the corresponding author.

## Appendix A

**Table A1:** Rank ordering of “top strategies”.

Criteria	Description	Ranking Principle
1. Success of the Strategy in Addressing Challenging Behavior	Teachers/aides report strategy success as successful, unsuccessful, or not reported.	Higher rank for strategies with a lower percentage of unsuccessful reporting.
2. Number of Challenging Behaviors Observed Post-Intervention	Number of behaviors observed after the intervention was implemented (or timed marked as being implemented), including existing or additional challenging behaviors.	Higher rank for strategies with fewer challenging behaviors observed.
3. Number of Skills Observed	Number of skills observed after the intervention, including academic, developmental, motor, gross, adaptive, language, communication, and play/social skills.	Higher rank for strategies with more skills observed.
4. Heart Rate Reduction	Magnitude of heart rate reduction following the implementation of an intervention strategy.	Higher rank for strategies with a greater reduction in heart rate.
5. Time for the Child to Return to the Green Heart Rate Zone	Overall time taken for a child to return to their heart rate resting state baseline (green zone) following the intervention.	Higher rank for strategies with a shorter time to return to the green zone.
6. Time Since the Strategy was Employed	Consideration of the amount of time since the strategy was used.	Higher rank for strategies that have been more recently implemented.
Results	Strategies are sequentially assessed, progressing to the next criterion only if the preceding one is identical between strategies.	Strategies are rank ordered to determine the top 3 strategies for pop-up notifications.

Note. Green heart rate zone indicates when a child’s heart rate is at baseline. Heart rate reduction, observed challenging behaviors, and observed skills are measured in the 5 min succeeding the implementation of an intervention strategy.

**Table A2:** Observation guide (First 3 of 15 min).

Time Interval	Child Behavior	Intervention Strategies	Strategy Effectiveness	Notes
0–3 mins Time: ______-______	Challenging Behavior ☐ Aggression (towards another person) ☐ Climbing on furniture (at least 3 s) ☐ Dropping (body to floor) ☐ Escape behavior (fleeing) ☐ Loud noises (screaming) ☐ Negative peer interaction (not aggression) ☐ Noncompliance (defiance after directive) ☐ Property destruction (aggression towards objects/furniture) ☐ Self-injury (aggression towards self) ☐ Stripping (not in context) ☐ Swearing (to self or others) ☐ Other:	☐ Proactive Antecedent-Based Interventions Augmentative and Alternative Communication Class-Wide Behavior Management Cognitive Behavioral/Instructional Strategies Delay- and Denial-Tolerance Training Differential Reinforcement Discrete Trial Training Emotion-Regulation Training Feedback Functional Behavioral Assessment Functional Communication Training Homework Metaphors/Analogies Mindfulness-Based Strategies Modeling Naturalistic Intervention Prompting Reinforcement Role Play and Practice Self-Management Social Narratives Social Skills Training Time Delay (Prompting) Video Modeling Visual Supports ☐ Other:	Overall Proactive Strategies were: ☐ Effective ☐ Not effective ☐ Cannot determine ☐ N/A	
Activity				
IRR ____/21 _____%	Skill Acquisition ☐ Academic and developmental skills (e.g., numeracy, literacy, motor, sensory processing) ☐ Adaptive skills (e.g., personal care, independence) ☐ Language and communication (e.g., expressive or receptive language, or spontaneous communication) ☐ Play or social skills (e.g., pretending, peer interaction) ☐ Other:	☐ Reactive Emotion-Regulation Training Extinction Mindfulness-Based Strategies Modeling Naturalistic Intervention Physical Safety Management Prompting Response Interruption/Redirection Time Delay (Prompting) Visual Supports ☐ Other:	Overall Reactive Strategies were: ☐ Effective ☐ Not effective ☐ Cannot determine ☐ N/A	

## Appendix B

**Table A3:** Descriptive statistics of strategy effectiveness.

Effectiveness	Frequency	Percent
Missing	63	24.50
Effective	153	59.50
Ineffective	41	16.00
Total	257	100.00

**Table A4:** Binary logistic regression on the effect of heart rate reduction % and behavioral data on intervention strategy effectiveness, controlling for child.

	Model		Individual Predictors			95% C.I. for Exp(B)	
	***p***	***χ^2^***	***p***	***Exp(B)***	***B***	***Lower***	***Upper***
*Block*	<0.001	29.54					
HR Reduction %			0.101	1.05	0.05	0.468	1.10
Mins to Return to Green HR Zone			0.354	0.783	−0.24	0.991	1.31
Subsequent Behaviors			0.036	0.784	−0.24	0.625	0.985
Subsequent Skills			0.001	3.65	1.29	1.67	7.94
Child			0.009	3.63	1.29	1.38	9.58

**Note.** HR = Heart Rate. Green HR Zone is when a child’s heart rate is at baseline. Strategy Type coded so that 1 is reactive and 0 is proactive.

**Table A5:** Frequency and average heart rate reduction % of intervention strategies by strategy type.

Intervention Strategy by Strategy Type	Frequency	HR Reduction %
***Proactive***		
Antecedent-Based Intervention	37	18.33%
Augmentative and Alternative Communication	1	11.38%
Class-Wide Behavior Management	3	11.85%
Delay and Denial Tolerance Training	1	21.48%
Discrete Trial Training	1	22.83%
Emotion Regulation Training	7	26.08%
Feedback	9	24.65%
Functional Communication Training	9	15.05%
Metaphors/Analogies	1	16.81%
Mindfulness-Based Strategies	5	24.00%
Modeling	14	14.65%
Naturalistic Intervention	35	17.78%
Prompt Time Delay	1	26.60%
Prompting	35	16.16%
Reinforcement	22	16.96%
Self-Management	5	30.53%
	***N* = 186**	***M* = 18.24%**
***Reactive***		
Emotion Regulation Training	2	21.30%
Extinction	1	21.48%
Mindfulness-Based Strategies	1	22.68%
Modeling	3	14.33%
Naturalistic Intervention	3	14.63%
Physical Safety Management	3	3.14%
Prompting	9	18.07%
Response Interruption/Redirection	18	12.20%
	***N* = 40**	***M* = 14.13%**

**Table A6:** Binary logistic regression on the effect of strategy type, heart rate reduction %, and behavioral data on strategy effectiveness, controlling for child.

	Model		Individual Predictors			95% C.I. for Exp(B)	
	***p***	***χ^2^***	***p***	***Exp(B)***	***B***	***Lower***	***Upper***
*Block*	<0.001	34.18					
HR Reduction %			0.127	1.04	0.043	0.988	1.10
Mins to Return to Green HR Zone			0.331	0.773	−0.257	0.461	1.30
Subsequent Behaviors			0.054	0.796	−0.228	0.632	1.00
Subsequent Skills			0.001	3.61	1.28	1.66	7.89
Strategy Type (1)			0.031	3.19	1.16	1.11	9.17
Child			0.551	0.551	−0.101	0.647	1.26

**Note.** HR is an abbreviation for heart rate. Green HR Zone is when a child’s heart rate is at baseline. Strategy Type coded so that 0 is proactive and 1 is reactive.
